# Room temperature synthesis of cobalt-manganese-nickel oxalates micropolyhedrons for high-performance flexible electrochemical energy storage device

**DOI:** 10.1038/srep08536

**Published:** 2015-02-23

**Authors:** Yi-Zhou Zhang, Junhong Zhao, Jing Xia, Lulu Wang, Wen-Yong Lai, Huan Pang, Wei Huang

**Affiliations:** 1 Key Laboratory for Organic Electronics & Information Displays (KLOEID), Institute of Advanced Materials (IAM), National Jiangsu Synergetic Innovation Center for Advanced Materials (SICAM), Nanjing University of Posts and Telecommunications (NUPT), Nanjing. 210023, China; 2 Key Laboratory for Clearer Energy and Functional Materials of Henan Province, College of Chemistry and Chemical Engineering, Anyang Normal University, Anyang. 455000, China; 3 State Key Laboratory of Coordination Chemistry, Nanjing University, Nanjing. 210093, China

## Abstract

Cobalt-manganese-nickel oxalates micropolyhedrons were successfully fabricated by a room temperature chemical co-precipitation method. Interestingly, the Co_0.5_Mn_0.4_Ni_0.1_C_2_O_4_*nH_2_O micropolyhedrons and graphene nanosheets have been successfully applied as the positive and negative electrode materials (a battery type Faradaic electrode and a capacitive electrode, respectively) for flexible solid-state asymmetric supercapacitors. More importantly, the as-assembled device achieved a maximum energy density of 0.46 mWh·cm^−3^, a decent result among devices with similar structures. The as-assembled device showed good flexibility, functioning well under both normal and bent conditions (0°–180°). The resulting device showed little performance decay even after 6000 cycles, which rendered the Co_0.5_Mn_0.4_Ni_0.1_C_2_O_4_*nH_2_O//Graphene device configuration a promising candidate for high-performance flexible solid-state asymmetric supercapacitors in the field of high-energy-density energy storage devices.

High-performance energy conversion-storage devices have been receiving a lot of research attention recently^1^,^2^,^3^,^4^,^5^. Electrochemical capacitors, also known as supercapacitors (SCs), are widely regarded as suitable candidates for the next generation power source owing to their high power density, high stability and low fabrication cost^6^,^7^. SCs are widely applied in many fields such as emergency power supplies, electronic devices, and hybrid-electric machines. However, the energy density of SCs are generally not high enough to be used as sustainable power sources for many applications. Much effort has been devoted to improving the energy density of SCs. One promising strategy is to design nanostructured electrode materials with a large amount of active sites and high structural stability. Various micro/nanomaterials including nanoparticles^8^, nanowires^9^,^10^, nanotubes^11^ and nanosheets^12^,^13^ have been successfully applied as SC electrodes with high energy density. On the other hand, solid-state asymmetric supercapacitors (SASCs) comprising a battery type Faradaic electrode as an energy source and a capacitive electrode as a power source are promising alternatives to conventional SCs which are symmetrical using liquid electrolytes^14^,^15^,^16^,^17^,^18^. SASCs using solid-state electrolytes can avoid the potential problem of electrolyte leakage and are more environmentally friendly. What's more, compared with conventional SCs, SASCs have other advantages including light-weight, small-size, good reliability, ease of handing, and a wider of operating temperature range^19^,^20^, making SASCs suitable for wearable and flexible electronics. As an example, Lu et al have successfully reported a SASC based on MnO_2_//Fe_2_O_3_ nanowires^21^. However, it still remains a great challenge to fabricate high-performance SASCs.

Transition metal oxalate micro/nano materials have been recently explored as precursors for the synthesis of porous transition metal oxides (NiO^22^,^23^, Mn_2_O_3_^22^, CeO_2_^24^ and so on) and porous mixed transition metal oxides (NiMn_2_O_4_^25^, Ni_0.3_Co_2.7_O_4_^26^,^27^, ZnO-NiO^28^, Co-Ni-Mn oxide nanowires^29^ and so on). As a matter of fact, however, metal oxalates themselves can be used as electrode materials. Compared with the synthesis of traditional metal oxides, the synthesis of the metal oxalates generally involves only simple synthetic methods in aqueous solution (usually, a simple and scalable co-precipitation method under room temperature) thus the materials are obtained with low cost, environmental friendliness and safety. Micro/nanostructural nickel oxalates are an interesting class and have been successfully applied to make electrodes for electrochemical capacitors^30^ and asymmetric supercapacitors^31^.

In this work, we have successfully synthesized uniform cobalt-manganese-nickel oxalates (Co_0.5_Mn_0.4_Ni_0.1_C_2_O_4_*nH_2_O) micropolyhedrons by a room temperature chemical co-precipitation method. The synthesis method is green with low energy consumption. More importantly, Co_0.5_Mn_0.4_Ni_0.1_C_2_O_4_*nH_2_O micropolyhedrons can be successfully used as the positive electrode materials for SASCs (graphene nanosheets as the negative electrode material). A maximum energy density of 0.46 mWh·cm^−3^ was obtained easily by the as-assembled SASC, which was higher than most of previous results. The good flexiblity of the as-assembled SASC device enabled it to work under both the normal and the bent condition (0°–180°). An efficiency cycle ability was found after 6000 cycles, which made Co_0.5_Mn_0.4_Ni_0.1_C_2_O_4_*nH_2_O//Graphene SASC a promising candidate for high-performance flexible SASC in the field of high-energy-density energy storage devices.

## Results

**Figure 1:**
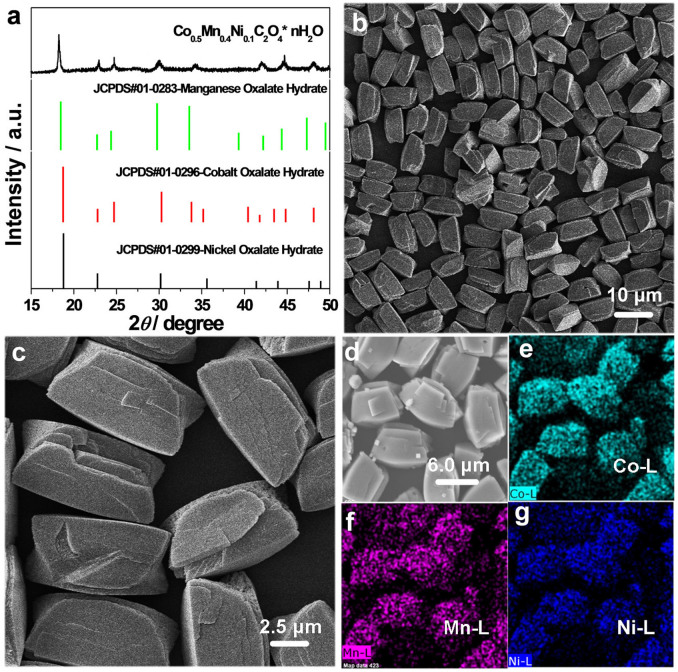
(a) XRD patterns of as-prepared samples, JCPDS#01-0296-cobalt oxalate hydrate, JCPDS#01-0283-manganese oxalate hydrate and JCPDS#01-0299-nickel oxalate hydrate; (b–d) SEM images, and (e–g) EDS-mapping images for different elements.

Co_0.5_Mn_0.4_Ni_0.1_C_2_O_4_*nH_2_O micropolyhedrons were obtained by a room temperature chemical co-precipitation condition (See experimental section). The XRD patterns of the as-prepared samples are shown in Figure 1. In view of the single oxalate phases (JCPDS#01-0296-cobalt oxalate hydrate, JCPDS#01-0283-manganese oxalate hydrate and JCPDS#01-0299-nickel oxalate hydrate), all the peaks of the as-prepared product are from the coupling result of three phases, which could not be indexed to a single oxalate. It indirectly indicates that the mixed oxalates have formed. Figure 1b,c show the typical SEM images of the as-prepared sample, and the uniform micropolyhedron with 10 μm was the main product. To identify the correct element ratio of the as-prepared sample, EDS-Mapping has been measured (Figure 1e–g). The contrast of light-shade intensity is related with the element content. Clearly, the order of the element content is Co > Mn > Ni from the results of Figure 1e–g. And the detailed quantitative calculation is also shown in Figure S1: C 12.0%, O 54.2%, Mn 12.1%, Co 16.2%, Ni 3.2%. The three metal element atom ratio is Co:Mn:Ni = 5:4:1, which is highly consistent with the raw material ratio. Additionally, Co, Mn and Ni contents were analyzed by ICP-OES (PE-3300DV) after the sample was dissolved. The ICP result confirmed the three element atom ratio was Co:Mn:Ni = 5:4:1, consistent with the EDS result.

## Discussion

**Figure 2:**
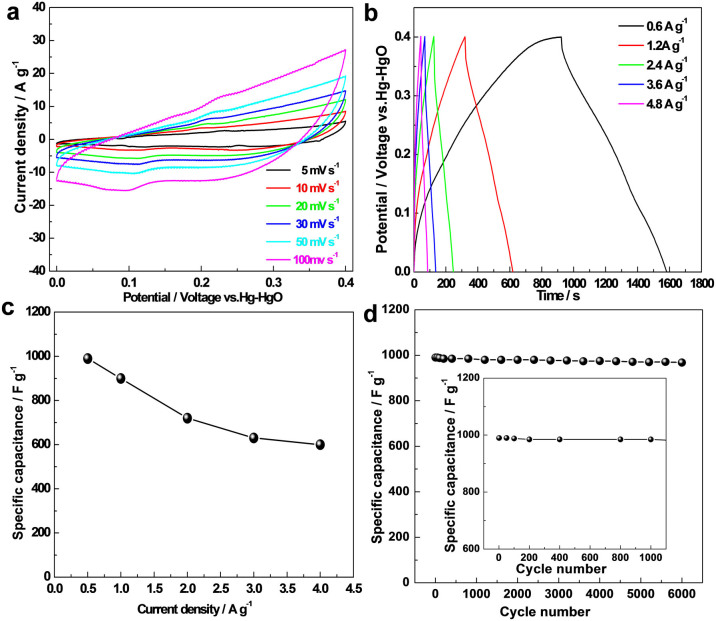
In a three-electrode system in 3.0 M KOH solution. (a) CV curves with different scan speeds; (b) CP curves with different current densities; (c) Specific capacitance calculated based on the discharge curve from (b), and (d) Cycling life test at 0.6 A·g^−1^.

The electrochemical property of the as-prepared Co_0.5_Mn_0.4_Ni_0.1_C_2_O_4_*nH_2_O electrode was first studied in the three electrode configuration by Cyclic Voltammetry (CV) and Chronopotentiometry (CP) measurements (Figure 2). From Figure 2a and Figure S2, unlike the shape of electric double-layer capacitance, CV curves suggested that the electrochemical capacity was mainly pseudocapacitive. What's more, the Faradaic pseudocapacitive characteristics may result from the redox mechanism of the surface metal ion. A reversible redox reaction was proposed to occur on the as-prepared electrode:
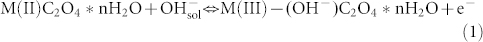


CP curves of the as-prepared electrode with different current densities are shown in Figure 2b. Clearly, the discharge time decreased with the increase of current density. However, it exhibited rather high electrode polarization even at low current densities as shown in Figure 2b, we thus supposed that irreversible reactions happened. The specific capacitances calculated from the discharging curves with different current densities are shown in Figure 2c. The specific capacitances achieved 990 F·g^−1^ at 0.6 A·g^−1^, and 600 F·g^−1^ at even 4.0 A·g^−1^ in 3.0 M KOH solution. Interestingly, the as-prepared electrode exhibited stable cycling performance with a specific capacitance of 968 F·g^−1^ even after 6000 cycles (Figure 2d). Clearly, the as-prepared electrode showed no capacity decay after 1000 cycles in the inset of Figure 2d, further confirming the pragmatic value of the Co_0.5_Mn_0.4_Ni_0.1_C_2_O_4_*nH_2_O electrode.

The graphene electrode in three-electrode system have been evaluated. As depicted in Figure S3, the graphene electrode shows a good specific capacitance (277 F g^−1^, 0.6 A g^−1^) which also offers a good cycle life (88.8%, Retention 10000 cycle).

**Figure 3:**
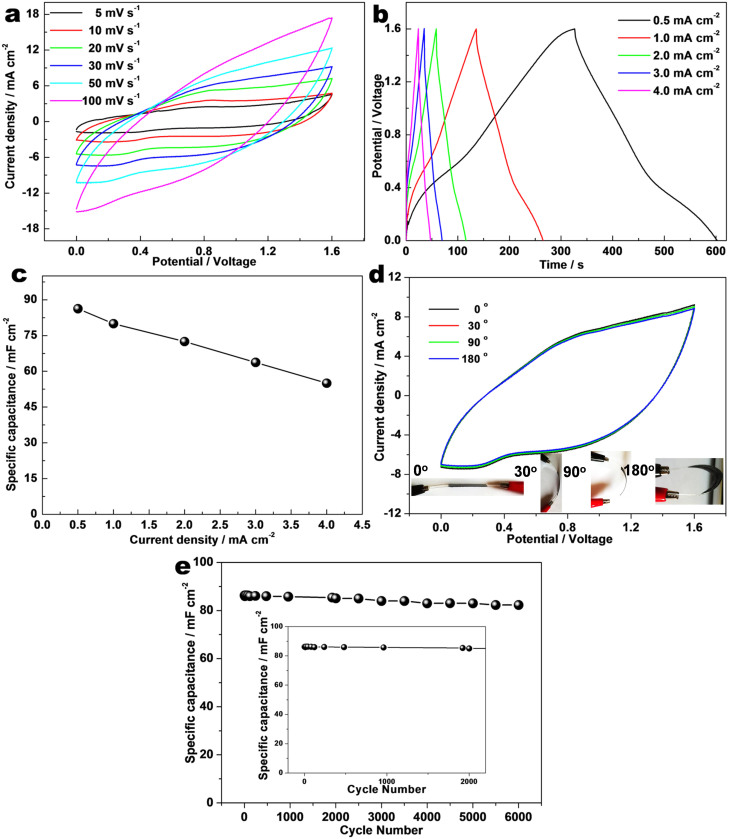
(a) Cyclic voltammetry of the as-prepared SASC device with different scan rate from 5 to 100 mV s^−1^; (b) The galvanostatic charge-discharge curves with different current densities, 0.50–4.0 mA·cm^−2^; (c) Corresponding specific capacitance calculated by discharge curves in (b); (d) CV curves at a scan rate 30 mV s^−1^ with different bended degrees (0°, 30, 90° and 180°, Inset-corresponding optical images), and (e) Cycle life testing at 0.5 mA·cm^−2^ for 6000 cycles, and 2000 cycles-Inset.

We have successfully assembled a SASC device using Co_0.5_Mn_0.4_Ni_0.1_C_2_O_4_*nH_2_O micropolyhedrons and graphene nanosheets. To measure the electrochemical property of the as-prepared device, CV and CP measurements were tested carefully. Unlike the three-electrode electrochemical feature, the SASC device displayed a quasi-rectangular CV geometry with feeble redox peaks, indicating a combination of both pseudocapacitive and electric double-layer capacitor properties at all scan rates in Figure 3a. Moreover, galvanostatic charge-discharge curves of the as-prepared device with different current densities (0.50–4.0 mA·cm^−2^) are shown in Figure 3b. The good symmetry of curves showed excellent reversibility of the as-prepared device. The linear sloping of galvanostatic charge-discharge curves is not only characteristic of EDLC, but also of the combination the pseudocapacitive behavior with EDLC behavior. In other word, the electrochemical property of the SASC device results from the combination of a battery type Faradaic electrode and a capacitive electrode. The largest specific capacitance of the as-prepared device can reach up to 86.3 mF·cm^−2^ at a current density of 0.50 mA·cm^−2^, 55 mF·cm^−2^ at 4.0 mA·cm^−2^ in Figure 3c. In order to test the flexibility of the as-prepared device, the SASC was bended with different angles (0°, 30°, 90°, and 180°, Inset-corresponding optical images), while corresponding CV tests were carried out (Figure 3d). Remarkably, throughout the bending processes, the shape of CV curve was nearly unchanged, suggesting good flexibility of the device. In fact, we have measured the performance of as-prepared device after 400 bending times as depicted in Figure S4. The as-prepared device showed only 0.1% performance decay after 400 bendings. The SASC maintained 98.6% of the initial specific capacitance after 2000 cycles and at least 98.6% after 6000 cycles as shown in Figure 3e. The stable cycling performance is much better than most previous results, such as PANI/CNT//PANI/CNT (88.6% after 1000 cycles)^17^, MnO_2_ NW/graphene//graphene (79% after 1000 cycles)^32^, and RuO_2_-graphene//graphene (95% after 2000 cycles)^33^. In addition, the electrochemical impedance spectroscopy (EIS) analysis before and after the cycling are compared in Figure S5. From the EIS result in Figure S5, the resistance only changed slightly, which further comfirms the stability of the electrochemical performance.

**Figure 4:**
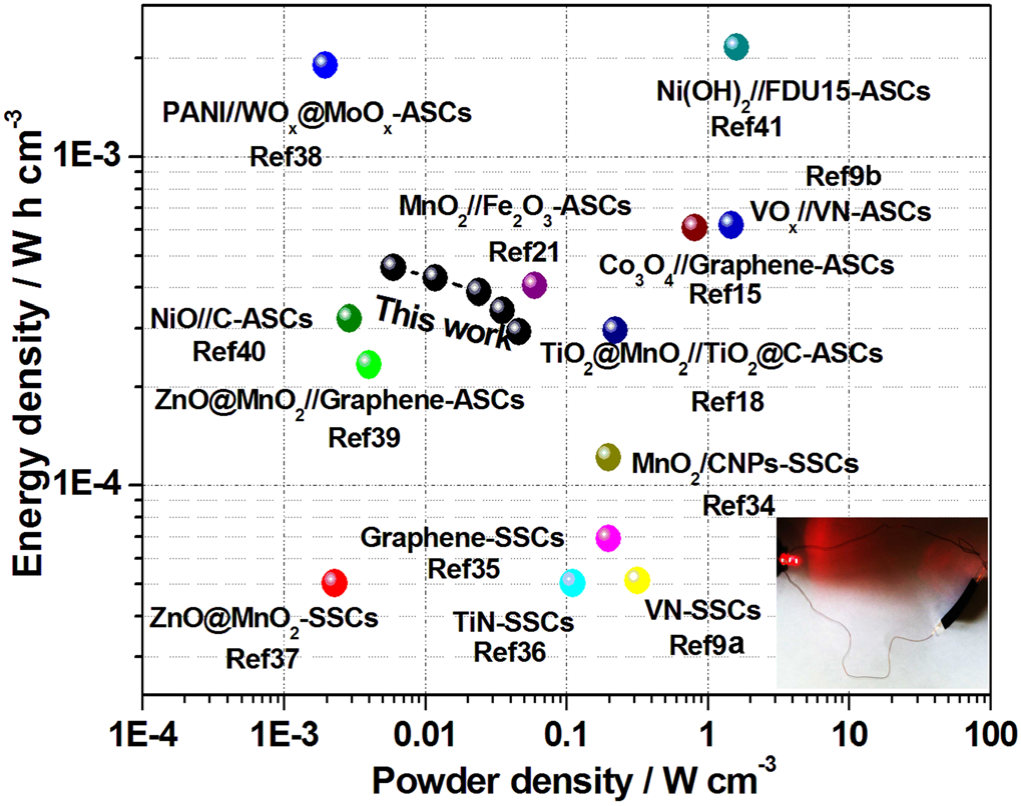
Ragone plots of the as-prepared SASC device. The values reported for other previous devices are added for comparison^9^,^15^,^18^,^21^,^34^,^35^,^36^,^37^,^38^,^39^,^40^,^41^. Inset shows a red LED (1.5 V) powered by the as-prepared SASC.

The volumetric energy and power densities of the as-prepared flexible SASC calculated based on the data in Figure 3b are shown in Figure 4. For comparasion, the volumetric power and energy densities of other energy storage devices are also plotted. The as-fabricated flexible SASC possessed a maximum volumetric energy density of 0.46 mW·h·cm^−3^ at 0.5 mA·cm^−2^, and 0.29 mW·h·cm^−3^ at 4.0 mA·cm^−2^, also showing good rate performance of the flexible SASC device. Moreover, the maximum volumetric energy density of the as-prepared flexible SASC was considerably higher in comparison with those of recently reported devices^9^,^18^,^21^,^34^,^35^,^36^,^37^,^39^,^40^, such as TiO_2_@MnO_2_//TiO_2_@C (0.5 mA·cm^−2^–0.30 mW·h·cm^−3^)^18^, MnO_2_//Fe_2_O_3_ (0.5 mA·cm^−2^–0.41 mW·h·cm^−3^)^21^ and ZnO@MnO//Graphene (0.5 mA·cm^−2^–0.234 mW·h·cm^−3^)^39^. However, the obtained maximum volumetric energy density was lower than those from ref. 9b, 15, 38, 41 and 42. Additionally, the SASC device can offer a maximum power density of 46 mW·cm^−3^ at 4.0 mA·cm^−2^, which is much higher than that of recently reported ZnO@MnO_2_^37^, polyaniline//WO*_x_*@MoO*_x_*^38^, and ZnO@MnO_2_//Graphene^39^, and NiO//C^40^, but lower than that of other devices^9^,^15^,^18^,^21^,^34^,^35^,^36^,^41^,^42^. The results above confirmed that the Co_0.5_Mn_0.4_Ni_0.1_C_2_O_4_*nH_2_O micropolyhedron is a promising anode material for SASCs. To demonstrate the potential application of the as-prepared ASC device, an ASC device was employed to power a red light-emitting-diode (LED) as shown in the inset of Figure 4. The ASC device can power a red LED (1.5 V) for about 2 min after charging at 0.5 mA cm^−2^ for 30 s.

We attribute the excellent electrochemical energy storage behavior to the desirable synergy of composition and nanostructure of as-prepared materials. Specifically, the primary nanopores (Figure S6) provide high electrochemical activity and relatively high active surface area (89 m^2^ g^−1^), while the secondary micropolyhedrons in micrometer dimensions prevent the undesirable agglomeration and ensure the stability of the porous structure. It is worth mentioning that the abundant mesopores are of great significance for the electrochemical processes.

In summary, Co_0.5_Mn_0.4_Ni_0.1_C_2_O_4_*nH_2_O micropolyhedron has been successfully synthesized *via* a room temperature chemical co-precipitation method. More importantly, a flexible SASC device has been successfully constructed with using the resulting Co_0.5_Mn_0.4_Ni_0.1_C_2_O_4_*nH_2_O micropolyhedrons and graphene nanosheets. The assembled Co_0.5_Mn_0.4_Ni_0.1_C_2_O_4_*nH_2_O//graphene SASC achieved a maximum energy density of 0.46 mWh·cm^−3^, which was higher than most of the reported solid state SCs. The resulting SASC exhibited excellent efficiency cycle stability for 6000 cycles, which rendered it as one of the top high-performance flexible solid-state asymmetric supercapacitors. Other applications are anticipated by fully exploiting the advantages of flexibility and high energy density originated from both the materials architecture and the novel design of the device. Further work is undergoing in our lab to improve the device performance and to extend the philosophy in this work into other systems within the framework of flexible/stretchable energy devices.

## Methods

### Synthesis of the Co_0.5_Mn_0.4_Ni_0.1_C_2_O_4_*nH_2_O micropolyhedron

All of the chemical reagents were of analytical grade and used without further purification. In a typical preparation, 10.0 mL 0.10 M Co(CH_3_COO)_2_-ethylene glycol solution, 8.0 mL 0.10 M Mn(CH_3_COO)_2_-ethylene glycol solution and 2.0 mL 0.10 M Ni(CH_3_COO)_2_-ethylene glycol solution were mixed and stirred for 10 min. Then 40.0 mL 0.10 M (NH_4_)_2_C_2_O_4_-H_2_O solution was added into the above solution, and then mixed vigorously for 2 h, and the resulting mixture was incubated at room temperature for 1 h. The resulting pink precipitate was collected by centrifugation, washed with water, ethanol several times and finally dried in air.

### Preparation of graphite oxide

GO was produced from natural graphite powders (universal grade, 99.985%) according to Hummers method. Firstly, natural graphite powders were treated by 5% HCl twice, then filtered, washed with distilled water thoroughly, and dried at 110°C for 24 h. Secondly, graphite powders (10 g) were placed in cold (0°C) concentrated H_2_SO_4_ (230 mL). KMnO_4_ (30 g) was added gradually with stirring and cooling. The temperature of the solution was not allowed to go up to 20°C. The mixture was stirred for 40 min, and distilled water (460 mL) was added slowly to an increase in temperature to 98°C. The temperature was held at 35 ± 3°C for 30 min. Finally, distilled water (1.4 L) and 30% H_2_O_2_ solution (100 mL) were added after the reaction. The solution was kept at room temperature for 24 h and then the mixture was filtered, washed with 5% HCl aqueous solution until sulfate could not be detected with BaCl_2_. The reaction product was dried under vacuum at 50°C for 24 h.

### Preparation of functionalized graphene sheets

The dried GO was thermally exfoliated at 300°C for 5 min under air atmosphere. The obtained samples were subsequently treated at 700°C in Ar for 3 h with a heating rate of 2°C/min.

### Characterizations

The morphology of as-prepared samples was observed by a JEOL JSM-6701F field-emission scanning electron microscope (FE-SEM) at an acceleration voltage of 5.0 kV. The phase analyses of the samples were performed by X-ray diffraction (XRD) on a Rigaku-Ultima III with Cu K_α_ radiation (*λ* = 1.5418 Å). Mn, Co and Ni contents were analyzed by ICP-OES (PE-3300DV) after the sample was dissolved.

### Fabrication and electrochemical study on the Co_0.5_Mn_0.4_Ni_0.1_C_2_O_4_*nH_2_O micropolyhedron electrode in a conventional three-electrode system

All electrochemical performances were carried out on Arbin-BT6000 electrochemical instrument in a conventional three-electrode system equipped with platinum electrode, a Hg-HgO as counter and reference electrode, respectively. Before electrochemical measurement, we have purged out O_2_ from the solution by the inert gas-Ar. The working electrode was made from mixing of active materials-the Co_0.5_Mn_0.4_Ni_0.1_C_2_O_4_*nH_2_O micropolyhedron electrode, acetylene black, and PTFE (polytetrafluoroethylene) with a weight ratio of 80:15:5, coating on a piece of nickel foam of about 1 cm^2^, and pressing it to a thin foil at a pressure of 5.0 MPa. The typical mass load of electrode material was 5.0 mg. The electrolyte was 3.0 M KOH solution. Galvanostatic charge–discharge methods were used to investigate capacitive properties of the Co_0.5_Mn_0.4_Ni_0.1_C_2_O_4_*nH_2_O micropolyhedron electrode, which were all carried out with an Arbin-BT6000 electrochemical instrument. Cyclic voltammetry measurements of the Co_0.5_Mn_0.4_Ni_0.1_C_2_O_4_*nH_2_O micropolyhedron electrode was conducted by using PARSTAT2273.

### Fabrication and electrochemical study of the flexible Co_0.5_Mn_0.4_Ni_0.1_C_2_O_4_*nH_2_O//graphene SASC

The PET substrates were first deposited with a layer of Pt film (~3 × 5 nm thick) and then coated with the slurry containing the active materials (Co_0.5_Mn_0.4_Ni_0.1_C_2_O_4_*nH_2_O micropolyhedrons or Graphene nanosheets) *via* a similar process to that in the three electrode system and were used as the working electrode after drying. Caution: Graphene nanosheets electrode was recoated for 6 times with the above graphene slurry. The ratio of the mass of the positive electrode to that of the negative electrode is 1:18. In the meantime, the PVA/KOH gel electrolyte was prepared as follows: the gel electrolyte (1.52 g PVA, 2.13 g KOH, and 15 mL DI water) was prepared at 75°C for 30 min under vigorous stirring. Subsequently, two pieces of such electrodes were immersed in the PVA/KOH gel solution for 5 ~ 10 min to adsorb a layer of solid electrolyte. After the excess water was vaporized, two pieces of such electrodes containing electrolyte were pressed together on a sheet out roller. Thus, the stacked SASC was fabricated. CV measurements were carried out at 5, 10, 20, 30, 50 and 100 mV·s^−1^ on an electrochemical work station (PARSTAT-2273). The flexible Co_0.5_Mn_0.4_Ni_0.1_C_2_O_4_*nH_2_O//Graphene SASC was galvano statically charged and discharged at the current density of 0.5–4.0 mA·cm^−2^ on the Arbin-BT6000 electrochemical instrument. All the electrochemical measurements were conducted at room temperature.

## Author Contributions

Y.Z.Z., J.H.Z., J.X., L.L.W., H.G.F. and H.P. conceived and designed the experiments. Y.Z.Z., W.Y.L., H.P. and W.H. analyzed the measurements. H.P. wrote the manuscript in collaboration with all the authors.

## Supplementary Material

Supplementary Information Supplementary Information
