# Supplementary material for: Room temperature synthesis of cobalt-manganese-nickel oxalates micropolyhedrons for high-performance flexible electrochemical energy storage device
Source: Sci Rep. 2015 Feb 23;5:8536. doi: 10.1038/srep08536 (PMC4336938; doi:10.1038/srep08536)
Supplement: Supplementary Information [file srep08536-s1.pdf]

Supplementary Information

Correspondence and requests for materials should be addressed to W.-Y. L. (iamwylai@njupt.edu.cn), H. P. (huanpangchem@hotmail.com) or W. H. (iamwhuang@njupt.edu.cn)

Room temperature synthesis of cobalt-manganese-nickel oxalates micropolyhedrons for high-performance flexible electrochemical energy storage device

Yi-Zhou Zhang,[1,2] Junhong Zhao,[1] Jing Xia,[1] Lulu Wang,[1] Wen-Yong Lai,*[2] Huan Pang*[1,2,3] and Wei Huang*[2]

[1] Key Laboratory for Clearer Energy and Functional Materials of Henan Province, College of Chemistry and Chemical Engineering, Anyang Normal University, Anyang, 455000, China. [2] Key Laboratory for Organic Electronics & Information Displays (KLOEID), Institute of Advanced Materials (IAM), National Jiangsu Synergetic Innovation Center for Advanced Materials (SICAM), Nanjing University of Posts and Telecommunications (NUPT), Nanjing, 210023, China. [3] State Key Laboratory of Coordination Chemistry, Nanjing University, Nanjing, 210093, China.

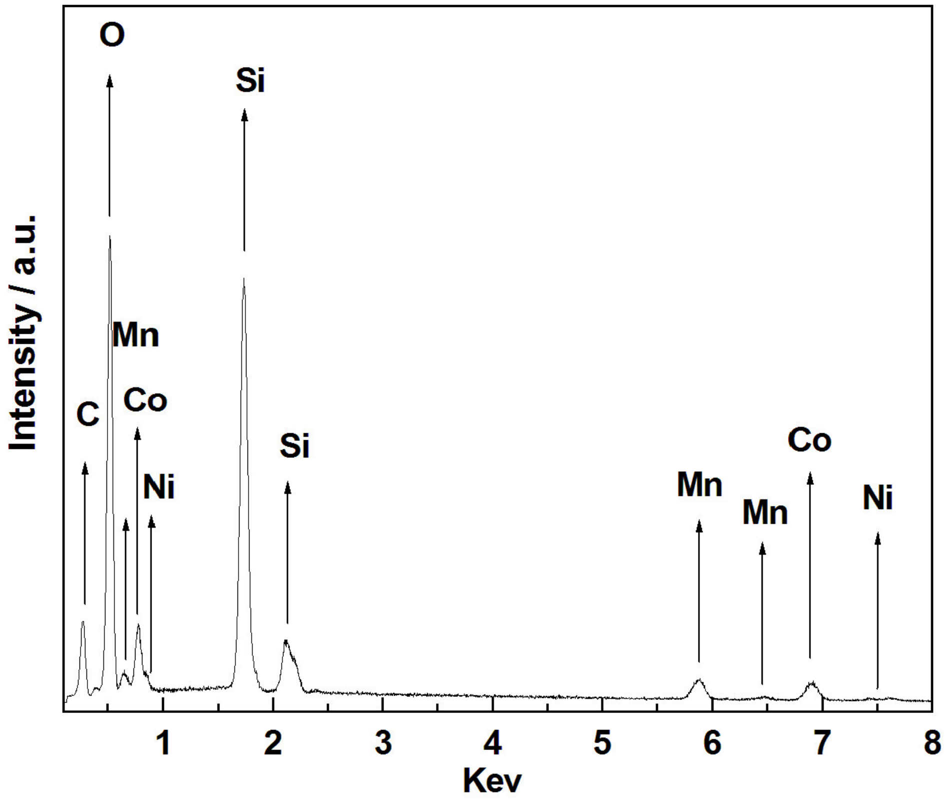

Figure S1. EDS patterns of the as-prepared samples.

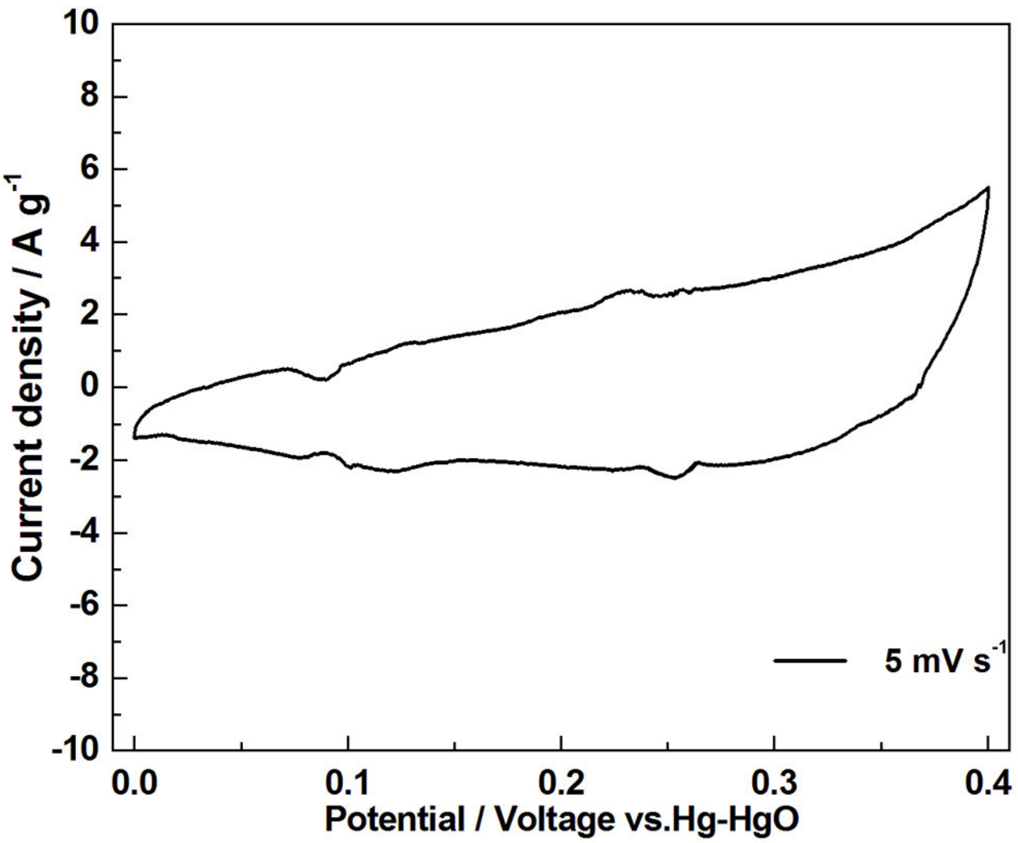

Figure S2. The CV curve in a three-electrode system in 3.0 M KOH solution of the as-prepared $Co_{0.5}Mn_{0.4}Ni_{0.1}C_2O_4*nH_2O$ micropolyhedron electrode at 5 mV s^{-1}.

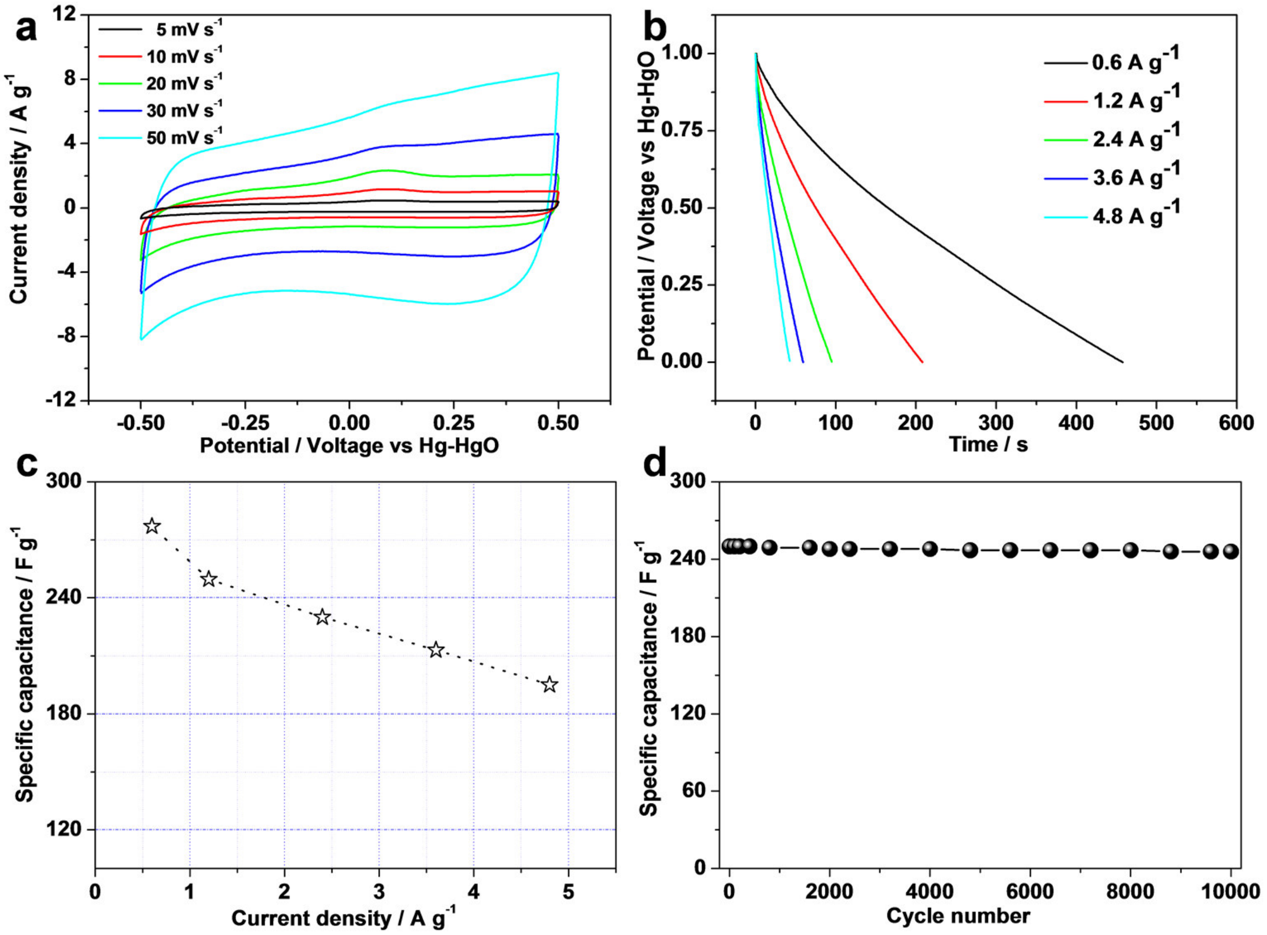

Figure S3. The graphene electrode in a three-electrode system in 3.0 M KOH solution: a) CV curves with different scan speeds, b) CP curves with different current densities, c) Specific capacitance calculated based on the discharge curve from b, and d) Cycling life test at 1.2 $A \cdot g^{-1}$.

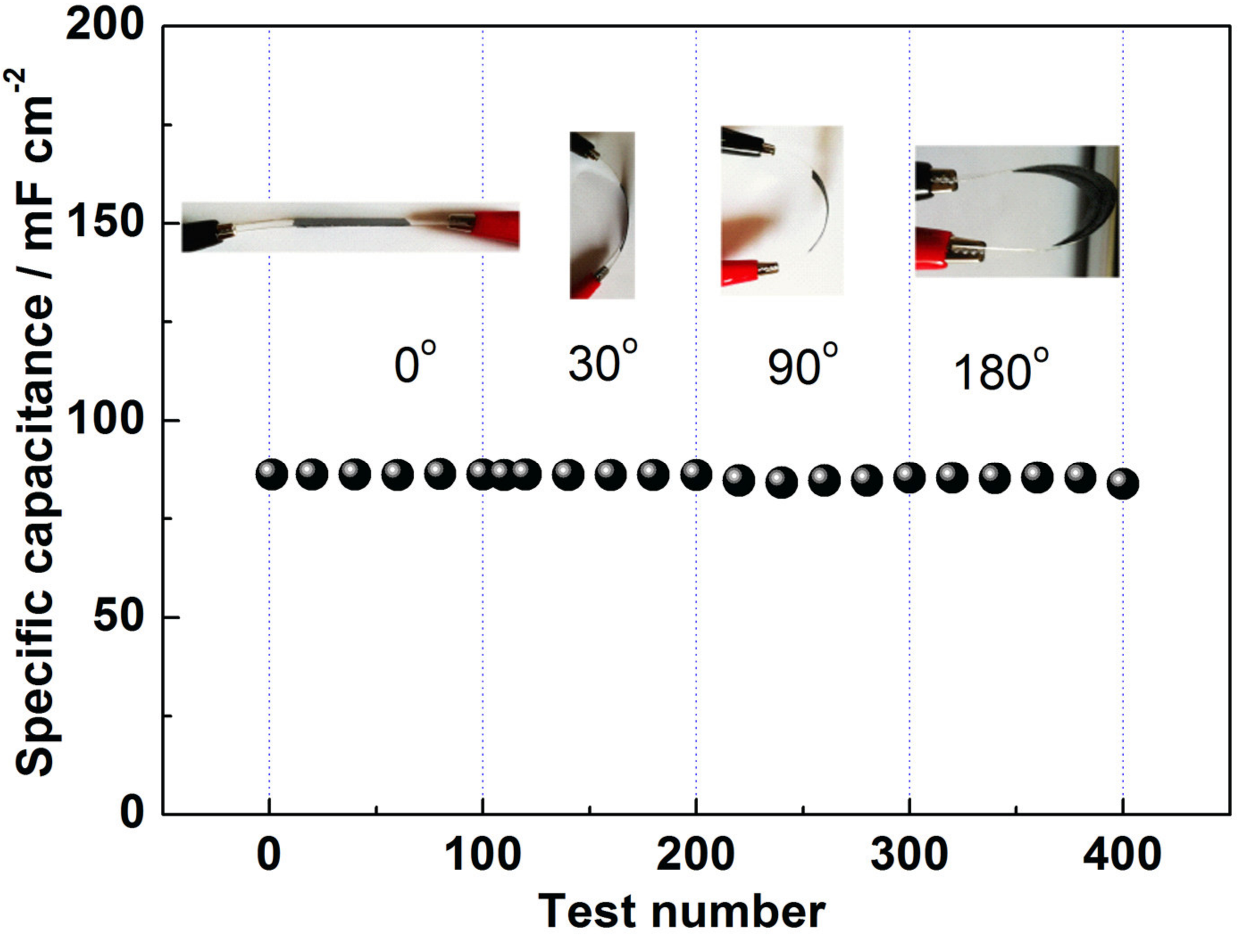

Figure S4. The specific capacitance of as-prepared device after 400 bending times with different bending modes.

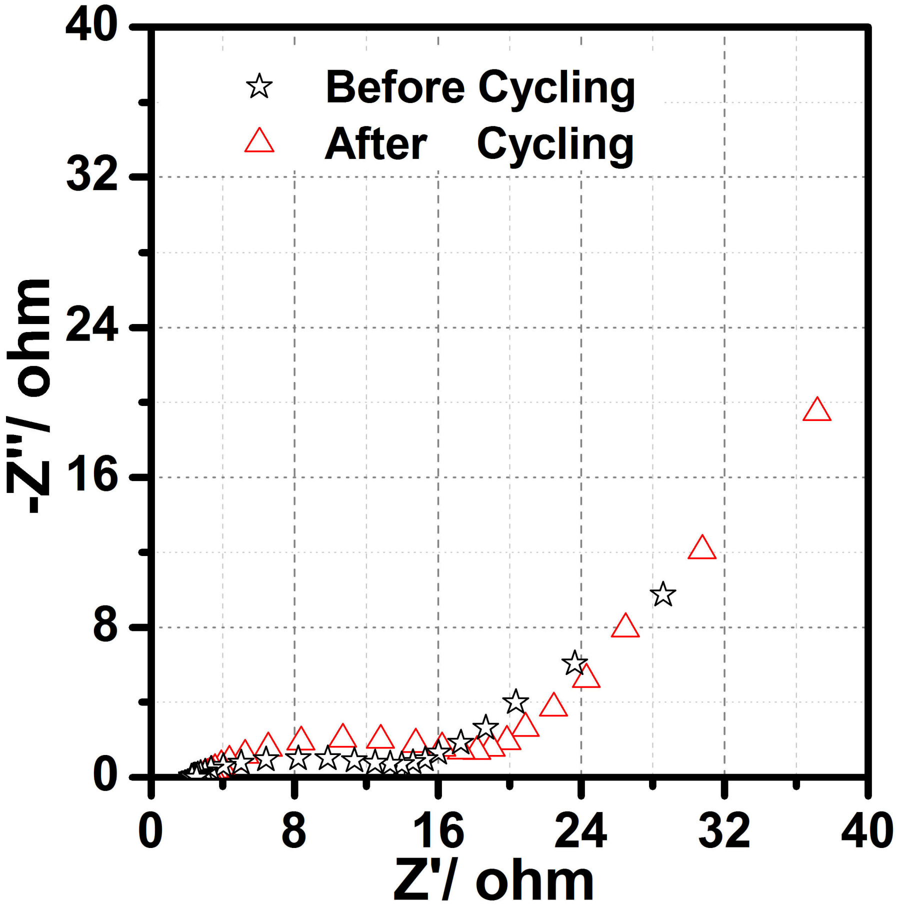

Figure S5. The electrochemical impedance spectroscopy (EIS) analysis of as-prepared SASC device before and after 6000 cycles.

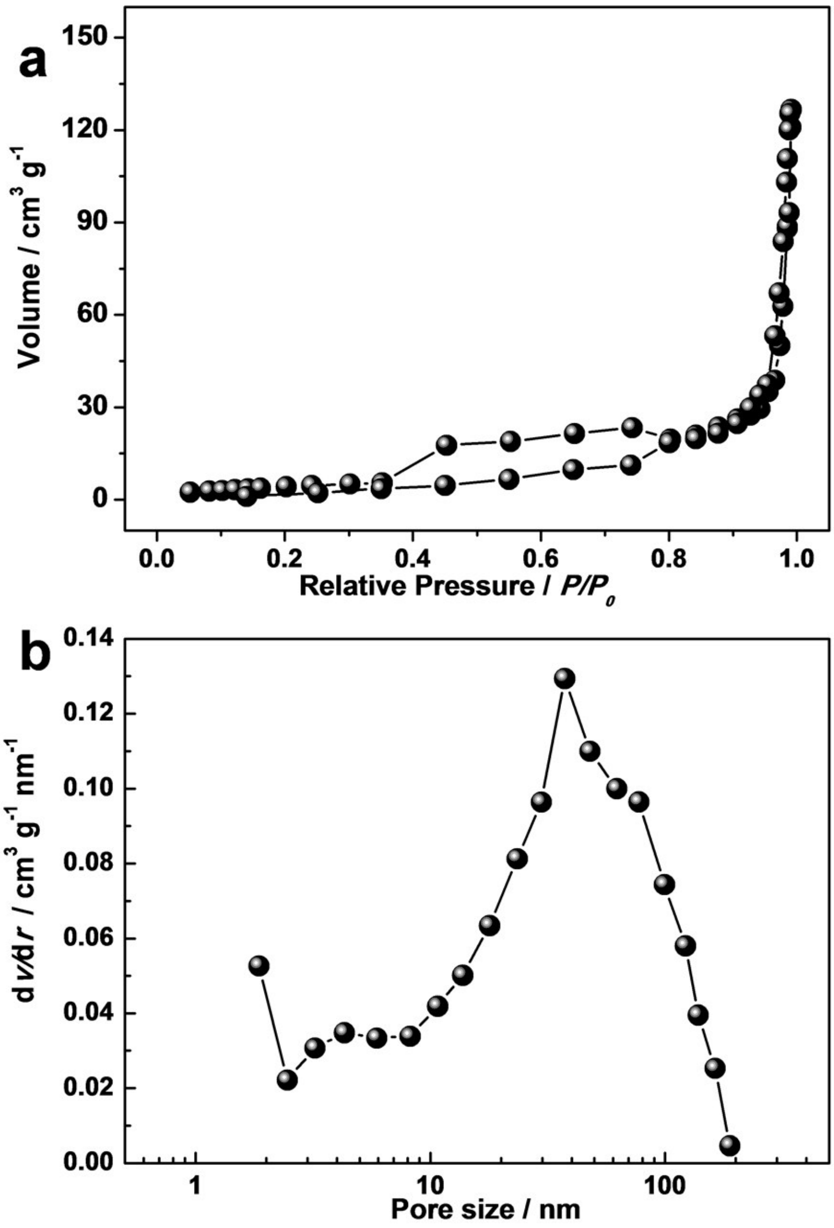

Figure S6 a) N_2 sorption isotherm, and b) Corresponding pore size distribution curve of as-prepared $Co_{0.5}Mn_{0.4}Ni_{0.1}C_2O_4$*$nH_2O$ micropolyhedrons.
